# Occurrence of *Helicobacter pylori* and its major virulence genotypes in dental plaque samples of patients with chronic periodontitis in Iran

**Published:** 2017

**Authors:** Sahel Valadan Tahbaz, Abbas Yadegar, Nour Amirmozafari, Siamak Yaghoobee, Mohammad Javad Ehsani Ardakani, Homayoun Zojaji

**Affiliations:** 1 *Department of Microbiology, Faculty of Basic Sciences, Research and Science Branch, Islamic Azad University, Tehran, Iran*; 2 *Foodborne and Waterborne Diseases Research Center, Research Institute for Gastroenterology and Liver Diseases, Shahid Beheshti University of Medical Sciences, Tehran, Iran*; 3 *Microbiology Department, School of Medicine, Iran University of Medical Sciences, Tehran, Iran*; 4 *Department of Periodontics and Implant, Faculty of Dentistry, Tehran University of Medical Sciences, Tehran, Iran*; 5 *Gastroenterology and Liver Diseases Research Center, Research Institute for Gastroenterology and Liver Diseases, Shahid Beheshti University of Medical Sciences, Tehran, Iran*; 6 *Basic and Molecular Epidemiology of Gastrointestinal Disorders Research Center, Research Institute for Gastroenterology and Liver Diseases, Shahid Beheshti University of Medical Sciences, Tehran, Iran*

**Keywords:** *Helicobacter pylori*, Chronic periodontitis, Virulence genotypes, Dental plaque, PCR

## Abstract

**Aim:**

This study was aimed to investigate the presence of *H. pylori* and its virulence genotypes in dental plaques of Iranian patients with chronic periodontitis.

**Background:**

*Helicobacter pylori* is a Gram-negative bacterium that is associated with atrophic gastritis, peptic ulcer, and gastric cancer. Several studies have detected this bacterium in the oral cavity, suggesting it as a potential reservoir.

**Methods:**

A hundred individuals were divided in 2 groups: 50 patients with chronic periodontitis (case group), and 50 subjects in non-periodontitis (control group). Supragingival and subgingival plaque samples were collected from the individuals using wood wedges and sterile paper points respectively, and prepared for PCR analysis.

**Results:**

Totally, *H. pylori* DNA was detected in 5 out of 100 (5%) dental plaques. Of 5 dental plaques positive for *H. pylori*, *cagA* gene was detected in 4 specimen, 3 in periodontitis group and one in non-periodontitis group. The *H. pylori vacA *s1m1 genotype was predominantly detected in 2/5 samples. The *babA2* gene was detected in all (5/5) *H. pylori*-positive dental plaques. There was no significant correlation between the presence of *H. pylori* genotypes from dental plaques and chronic periodontitis (*P* > 0.05).

**Conclusion:**

Our results revealed that the rate of *H. pylori* is very low in the dental plaques of Iranian patients with chronic periodontitis. Majority of *H. pylori* strains from oral cavity were highly virulent based on the main clinically virulence factors they carried.

## Introduction

*Helicobacter pylori* (*H. pylori*) is a spiral-shaped Gram-negative bacterium discovered by Warren and Marshall in 1982 (1). This highly prevalent organism is considered as the main risk factor for different gastroduodenal diseases such as chronic active gastritis, peptic ulcers and gastric cancer (2, 3). Several studies have reported the presence of *H. pylori* in dental plaque and saliva, implicating the oral cavity as a potential extragastric reservoir for *H. pylori *that may lead to reinfection of the stomach after eradication therapy (4-6). However, it is still unclear whether the oral cavity is a permanent or transient reservoir of this bacterium. Some researchers believe that *H. pylori *may be regarded as a member of the microbiota of the human oral cavity maintaining a commensal interplay with the host (7-9). In contrast, other authors have suggested that *H. pylori *only transiently colonizes oral cavities due to ingestion of contaminated foods or as a result of occasional gastroesophageal reflux (10, 11).

Genetic studies show that *H. pylori *strains display high variability in the gene content, and extreme heterogeneity has been observed among strains from different geographical regions (12, 13). Several *H. pylori* virulence factors have been identified that are associated with severe clinical outcomes. The cytotoxin associated antigen (*cagA*) gene is the most studied virulence factor of *H. pylori*, located at the end of an approximately 40 kb cluster of genes called *cag *pathogenicity island (*cag*PAI) (14, 15). The *cagA* gene is present in 60-70% of *H. pylori *strains based on the geographical origin, and is associated with severe gastroduodenal diseases including atrophic gastritis, peptic ulcer and gastric adenocarcinoma (16, 17).

The vacuolating cytotoxin (*vacA*) gene is another important virulence factor of *H. pylori*, which is supposed to be associated with the risk of developing peptic ulcers and gastric cancer (18, 19). The *vacA* gene is present in all *H. pylori* strains and has a mosaic structure consisting of allelic variations in its signal (s) and mid (m) regions, each having two distinct alleles (s1/s2, m1/m2) with different biological activities (20, 21). Strains with *vacA* s1m1 genotype produce large amounts of toxin and are strongly associated with a higher degree of inflammation and epithelial damage in the gastric mucosa; s1m2 strains produce moderate amounts of toxin while the s2m2 strains produce very little or no toxin (20).

In addition to the afore-mentioned factors, the *babA2* gene encodes a blood group antigen-binding adhesion (*babA*), which has been shown to mediate adherence of *H. pylori* to human lewis^b^ blood group antigens on gastric epithelial cells. Several studies suggested that the presence of *babA2* was associated with an increased risk of peptic ulcers and gastric cancer in Western populations (22, 23).

Dental plaque is a complex well-organized polymicrobial community that forms a biofilm in which several different bacterial species are intimately associated with each other and with the solid substratum through specific interactions (24, 25). Insufficient oral hygiene measures enhance the formation of dental plaque on the supragingival and subgingival tooth surfaces, where tissue destruction results in progressive deepening of the periodontal pockets (26, 27). Several studies have reported the presence of *H. pylori* in patients with gingivitis or chronic periodontitis, suggesting that progression of periodontal pocket and inflammation may favor colonization by this pathogen (28-30). *H. pylori* has also been proposed to have a special preference for the activated state of inflammation in periodontitis, and many studies have reported that carriage of *H. pylori* may be associated with periodontal disease (27, 29, 30).

Several studies have reported that *H. pylori* infection is common in Iran as in other developing countries (31-34). However, there are a few reports about the prevalence of *H. pylori* in the oral cavity of Iranian patients with chronic periodontal disease. Since poor oral hygiene measures are strongly associated with inflammatory responses in the periodontal disease, it seems biologically reasonable to investigate the presence of *H. pylori* in relation to periodontal conditions (28). Moreover, the prevalence and distribution of virulence genotypes of *H. pylori* strains in the oral cavity has been rarely investigated. Therefore, in this study we aimed to investigate the occurrence of *H. pylori* in dental plaque samples of individuals with or without chronic periodontitis, and also to determine the related virulence genotypes including *cagA*, *vacA* subtypes and *babA2* in an Iranian population.

## Methods

**Study population and sample collection**

The population of this study consisted of 50 patients with chronic periodontitis (case group), and 50 individuals without periodontal disease (control group) whom referred for treatment to Department of Periodontics and Implant of Tehran University of Medical Sciences, between September and December 2014. Written informed consent was obtained from all individuals in order to be included in the study. The study protocol was approved by the scientific research ethical committee of Tehran University of Medical Sciences.

Supragingival plaque samples were collected from both groups, control and case, using sterile wood wedges by scrapping the posterior tooth surfaces. Cotton rolls and air-drying with syringe were used to avoid saliva contamination of the plaque samples. Sterile paper points were also used to collect subgingival plaque samples from all patients with periodontal disease (case group). Subgingival plaque was obtained after removal of supragingival plaque by inserting two sterile paper points into the gingival sulcus/pocket for 20 seconds. Subgingival sampling sites in periodontitis patients exhibited clinical probing depth ≥5 mm and bleeding on probing. Each sample was immediately placed in a sterile tube containing 0.5 ml of Tris-EDTA buffer and mixed well by vortexing for 30-60 seconds. All samples were then immediately placed in coolers containing dry ice, transported to the laboratory, and stored at -20° C until DNA extraction.

**Preparation of Genomic DNA**

To prepare DNA from dental plaques, the samples were thawed at ambient temperature, and centrifuged at 12,000 × g for 15 min. The supernatant was discarded and the precipitate containing nucleic acids was kept on ice and used for DNA extraction. Then DNA was extracted from each sample using QIAamp DNA Mini Kit (Qiagen, Hilden, Germany) according to manufacturer’s instructions. The concentration and quality of DNA preparations were determined using a NanoDrop spectrophotometer, by measuring absorbance at wavelengths of A260 and A280 nm and also by agarose gel electrophoresis.

**Table 1:** Details of oligonucleotide primer sequences used in this study

Target gene	Primer designation	Oligonucleotide sequence (5΄- 3΄)	Annealing temperature (°C)	PCR product (bp)
16S rRNA	C97-20 H3A-20	GGCTATGACGGGTATCCGGC GCCGTGCAGCACCTGTTTTC	58	764
*ureC *fragment (*glmM*)	GlmM2-F GlmM1-R	GGATAAGCTTTTAGGGGTGTTAGGGG GCTTACTTTCTAACACTAACGCGC	56	296
*cagA*	93089 93261	AATACACCAACGCCTCCAAG TTGTTGCCGCTTTTGCTCTC	57	400
*vacA* s1/s2	VA1-F VA1-R	ATGGAAATACAACAAACACAC CTGCTTGAATGCGCCAAAC	57	259/286
*vacA* m1/m2	VAG-F VAG-R	CAATCTGTCCAATCAAGCGAG GCGTCAAAATAATTCCAAGG	57	570/645
*babA2*	bab7-F bab7-R	CCAAACGAAACAAAAAGCGT GCTTGTGTAAAAGCCGTCGT	52	271

**Table 2:** Demographic characteristics of patients with chronic periodontitis, and non-periodontitis

Demographic and clinical complication	Periodontitis group (*n* = 50) No. (%)	Non-periodontitis group (*n* = 50) No. (%)
Age (years)		
Mean Range	41.86 16-70	36.50 17-75
Gender		
Male Female	25 (50) 25 (50)	19 (38) 31 (62)
Antibiotic consumption		
Two months Six months	15 (30) 13 (26)	13 (26) 13 (26)
Smoking	9 (18)	1 (2)
Alcohol consumption	3 (6)	2 (4)
Gastritis	11 (22)	5 (10)
Dental scaling	8 (16)	9 (18)

**Table 3:** Frequency of* H. pylori* and its virulence genotypes in the dental plaque of chronic periodontitis patients (case group), and non-periodontitis group (control group

*H. pylori* genotypes	Periodontitis group (*n* = 50) No. (%)	Non-periodontitis group (*n* = 50) No. (%)	Total (*n* = 100) (%)
*H. pylori* in dental plaque			
Present Absent	4 (8) 46 (92)	1 (2) 49 (98)	5 (5) 95 (95)
*H. pylori* history in stomach			
Yes No	5 (10) 45 (90)	2 (4) 48 (96)	7 (7) 93 (93)
*cagA*	3 (6)	1 (2)	4 (4)
*vacA*s1 *vacA*s2	3 (6) 1 (2)	0 (0) 1 (2)	3 (3) 2 (2)
*vacA*m1 *vacA*m2	3 (6) 1 (2)	0 (0) 1 (2)	3 (3) 2 (2)
*vacA*s1m1 *vacA*s1m2 *vacA*s2m1 *vacA*s2m2	2 (4) 1 (2) 1 (2) 0 (0)	0 (0) 0 (0) 0 (0) 1 (2)	2 (2) 1 (1) 1 (1) 1 (1)
*babA2*	4 (8)	1 (2)	5 (5)

**PCR Amplification**

DNA extracted from dental plaque samples of all individuals was subjected to PCR assay using specific primers. To confirm the presence of *H. pylori* DNA in dental plaques, the *Helicobacter *genus- and species-specific PCR reactions were performed using primers for the 16S rRNA and *ureC* (*glmM*) genes (32). PCR-based genotyping was carried out on all *H. pylori*-positive DNA samples for the detection of *cagA* gene, *vacA* alleles (s1, s2, m1 and m2) and *babA2* in separate reactions using previously described primers (31, 32). The sequences of all oligonucleotide primers used in this study are listed in Table 1. All PCR reactions were performed in a 25 µl reaction mixture containing 5 µl of template DNA (approximately 100 ng), 0.1 mM of each primer, 2.5 µl of 10X PCR buffer, 100 mM of each dNTPs, 1 mM MgCl_2_, and 1.5 U of SuperTaq^TM^ DNA polymerase (HT Biotechnology Ltd., Cambridge, UK). PCR was performed in a thermocycler (Eppendorf, Hamburg, Germany) as follows: initial denaturation at 94°C for 4 min, which was followed by 35 cycles of denaturation at 94°C for 1 min, annealing at the indicated temperature for each reaction in Table 1 for 45 s, extension at 72°C for 1 min, and then final extension at 72°C for 10 min. Five-microlitre aliquots of the amplified products were analyzed by electrophoresis in a 1.2% (w/v) agarose gel (Roche Diagnostics, Mannheim, Germany) and stained with ethidium bromide (0.5 µg/ml). Stained amplicons were visualized on a UV transilluminator at 260 nm, and photographed (BioDoc-It System, UVP, USA). DNA extracted from *H. pylori* J99 (CCUG 47164) was included in every set of reaction as positive control. A no-template added reaction also served as negative control in each PCR experiment.

**Statistical analysis**

Statistical analysis of the data was performed using SPSS software version 21 (IBM Corporation, NY, USA). The Chi-square and Fischer’s exact tests were used to analyze the detection frequency of *H. pylori *in dental plaques of patients with and without periodontitis, and the possible association between *H. pylori *genotypes and periodontal disease. A *P *value of less than 0.05 was considered statistically significant.

## Results

**Prevalence of** ***H. pylori*** **in dental plaque samples**

Of 50 patients with chronic periodontitis (case group), randomly 25 (50%) were women and 25 (50%) were men with a mean age of 41.86 years (range 16 to 70 years). Among 50 individuals without periodontal disease (control group), 31 (62%) were women and 19 (38%) were men with a mean age of 36.5 years (range 17 to 75 years). Demographic characteristics of the patients with chronic periodontitis, and individuals without periodontal disease are presented in Table 2. Totally, *H. pylori* DNA was detected in 5 out of 100 (5%) dental plaque samples (Table 3). In the periodontitis group *H. pylori* DNA was detected in 4/50 (8%) dental plaque samples. *H. pylori* DNA was present in only one dental plaque sample from the individuals in non-periodontitis group. The prevalence of *H. pylori* in the dental plaques of patients with periodontitis was higher than in plaque samples from subjects without periodontal disease. However, no significant correlation was found between the presence of *H*. pylori in dental plaque and periodontal disease (*P *> 0.05).

**Virulence genotyping**

Of 5 dental plaque samples positive for *H. pylori*, the *cagA* gene was detected in 4 specimen, 3 in periodontitis group and one in non-periodontitis group (Table 3). The *H. pylori vacA* s1m1 genotype was detected in 2/5 samples, while the *vacA* s1m2, s2m1 and s2m2 genotypes all were present with the same prevalence (1/5) among the *H. pylori*-positive dental plaque samples. The *babA2* gene was detected in all (5/5) *H. pylori*-positive dental plaque samples, 4 in periodontitis group and one in non-periodontitis group. There was no significant correlation between *H. pylori* genotypes from dental plaque samples and chronic periodontitis (*P *> 0.05).

## Discussion

*H. pylori *infection is the most prevalent chronic bacterial disease affecting approximately half of the world’s population, and its prevalence shows wide geographical variations in different regions of the world. In developing countries, more than 80% of the population is infected that is inversely associated with socioeconomic status (2, 12). It is assumed that existence of *H. pylori* in the oral cavity, oral lesions and dental plaques may play an important role in the pathogenesis of reinfection. The presence of this organism may be very low within the mouth and the numbers of bacteria seem to vary in different niches in the oral cavity, such as dental plaque, tongue, and saliva as well as in oral ulcer lesions (35-39). There are many conflicting reports about the detection rate of *H. pylori *from dental plaques, ranging from 0-100% in different studies around the world (40). These noticeable variations in the prevalence of *H. pylori *in dental plaque samples basically arise from several methodological differences, such as differences in study designs, characteristics of the sample population, different methods used for plaque sampling, different assays exploited to identify the bacteria in dental specimen, and also differences in geographical regions.

The prevalence of *H. pylori* has been reported to be very low in dental plaque of Iranian patients with periodontal disease, and varied from 0 to 5.9% in recent studies (41, 42). In this study, we investigated the presence of *H. pylori* in the dental plaque samples of 100 participants enrolled, including 50 patients with chronic periodontitis and 50 individuals without periodontal disease. The supragingival and subgingival plaque samples were collected from these people using wood wedges and paper points, respectively. The results of the present study showed that *H. pylori* DNA was present totally in 5 (5%) dental plaque samples of all enrolled participants. We detected *H. pylori* DNA in 4 (8%) dental plaque samples of the patients with chronic periodontitis, and in only one dental plaque sample from the individuals in the non-periodontitis group. Our results are in agreement with the findings of previous studies from Iran which reported a very low prevalence of *H. pylori* in dental plaques among Iranian population (41, 42). Similar results have also been obtained from the previous studies conducted in South Africa, Brazil, Germany and Argentina where a low detection rate (1.7%, 2%, 5.4% and 10.2%, respectively) of *H. pylori* DNA was reported in dental plaques of the studied population (6, 43-45). On the other hand, our results are in contrast with the results of the other studies performed in some Asian countries such as Japan, India, Taiwan, Pakistan and China where a higher rate (38.3%, 42%, 43%, 51.6%, and 82.3%, respectively) of *H. pylori* infection was detected in dental plaques of the participants in the regions mentioned (46-50). Although the prevalence of *H. pylori *in dental plaque samples of patients with chronic periodontitis was higher than non-periodontitis group (4/50 versus 1/50, respectively), we did not find any significant correlation between the presence of *H. pylori *in dental plaque and periodontal disease.

*H. pylori cagA*-positive strains deliver the CagA oncoprotein into gastric epithelial cells in phosphorylated and non-phosphorylated forms, which promotes potent proinflammatory and proliferative responses associated with development of chronic gastritis and gastric cancer (51-53). The prevalence of *cagA* gene among *H. pylori* strains in Iranian population has been reported to vary from 62 to 94% in different studies (31, 33, 42, 54). In this study, 80% (4/5) of the dental plaque samples that were *H. pylori*-positive showed *cagA* genotype, which was in agreement with a recent study performed in Northern Brazil where 82% (58/71) of dental plaque specimen also exhibited *cagA* genotype (55). In addition, 75% (3/4) of *cagA*-positive dental plaque samples were observed in the periodontitis group, which was higher than non-periodontitis group with only one (1/4) *cagA*-positive sample. Our results were also consistent with a previous report from Iran by Momtaz et al. (56), who reported the* cagA* genotype in saliva samples with nearly the same prevalence (83%) as ours in plaque samples. However, our data was in contrast with another report from Brazil where the *cagA* gene was detected in 36.6% (11/30) of dental plaque samples (38).

It is presumed that gastric epithelial cell injury is caused by VacA cytotoxin, which induces multiple cellular effects such as host cell vacuolation, immune modulation and cell death. Additionally, the *vacA *alleles vary between *H. pylori* strains and these genotypic variations are associated with different risks for developing various clinical disorders of the upper gastrointestinal tract (21, 57). It has been reported that the *vacA *s1 and m1 genotypes are associated with higher degrees of lymphocytic and neutrophilic infiltrates, inflammation and severe *H. pylori*-related diseases, especially in Western countries, compared to that with *vacA *s2 and m2 strains that are rarely associated with peptic ulcer disease and gastric cancer (18, 58-61). The frequency of the *vacA *s1 and m1 genotypes in the Middle Eastern strains has been reported to be 71.5% and 32.8%, respectively, which is in concordance with our study (57). The *vacA* s1m1 genotype was predominantly detected in the *H. pylori*-positive dental plaque samples of patients with periodontitis (40%), which is in agreement with the results obtained in Northern Brazil (37%) (55). Meanwhile, *vacA* s1m2 and s2m1 genotypes were equally present in dental plaques of patients with periodontitis. However, the *vacA* s2m2 genotype that is believed to have low or non-cytotoxic activity was only found in the non-periodontitis group of the studied samples. Additionally, more than half of the *H. pylori*-positive plaque samples (60%) in this study that showed the *babA2* gene were simultaneously positive for the presence of *cagA* and *vacAs1* genotypes in patients with chronic periodontitis. This finding agrees with a previous report from Germany by Gerhard et al. (22), where they observed a significant association among *H. pylori* strains with triple positive genotypes, *vacAs1*, *cagA* and *babA2*. However, we did not find any significant association between the presence of this triple-positive genotype and chronic periodontal disease.

In conclusion, our findings revealed that the rate of *H. pylori* is very low in the dental plaques of Iranian patients with chronic periodontitis. Our results also showed that most of the *H. pylori *strains from oral cavity were highly virulent on the basis of the main clinically virulence factors they carried. In addition, the presence of the over-mentioned virulent genotypes of *H. pylori* strains in the dental plaque suggests that the oral cavity may serve as an important reservoir for reinfection, potentially affecting the eradication therapy of the *H. pylori*-associated gastroduodenal diseases. To our knowledge, this is the first study pursuing the detection of *H. pylori* DNA and its *cagA*, *vacA* and *babA2* virulence genotypes in dental plaques by using molecular methods in an Iranian population. However, future epidemiological research efforts with larger samples can help us to elucidate which variables influence the presence of *H. pylori* in the oral cavity and what are the clinical implications of virulence-associated genotypes in our understanding of the pathogenesis of *H. pylori* infection and related gastric disorders and especially chronic periodontal disease,

## Conflict of interests

The authors do not have any conflict of interest to be declared in the present study.
